# Gunshot Wounds of the Head: The Collective Experience of Surgeons

**Published:** 1915-02-27

**Authors:** 


### GUNSHOT WOUNDS OF THE HEAD.

### The Collective Experience of Surgeons.

The recent discussion on this subject at the Medical Society of London collected the experience and opinions of several well-known surgeons, and defined some principles to which it is necessary to give attention. In a more or less vague fashion it has been said that the military rifles of the present day are more humane than those formerly in use. The altered weight and shape of the bullet, and the greater velocity at which it travels, mean, of course, greater penetrating power, and there are numerous instances where a bullet has passed through a limb, leaving only a wound which has quickly healed. Similarly it has been noted that, in occasional instances at least, perforation of the abdomen or thorax has not proved inconsistent with recovery. That there is another side to this question was shown by an article published in The Hospital on December 19 last, where it was pointed out that in certain circumstances the modern bullet was able to inflict damage, even on the soft parts, which might be mistaken for the injuries produced by the so-called explosive bullet. The debate at the Medical Society has still further qualified the somewhat kindly view above referred to. As regards perforating wounds of the skull, it is significant that the only reference to them was made by a speaker who said none had come under his care, an experience which was "doubtless due to the fact that they are so frequently fatal."

One point much emphasised in the debate was the increase in intracranial pressure produced by these injuries. Sir Victor Horsley urged that the shock of the impact of the bullet was conveyed to the ventricles, largely through the agency of the cerebro-spinal fluid, and that in this way there resulted functional inhibition of the respiratory centre. When to this is added increase of pressure within the skull, as a consequence of hæmorrhage, an additional embarrassment to respiration is added. Hence the suggestion that prompt trephining and washing out of the blood in cases of this order might save life, and an instance was quoted in which this operation was successfully performed at a dressing station by the officer of the R.A.M.C. in charge. Another example of the added pressure within the cranial cavity following skull injuries was described by Mr. W. H. H. Jessop, who called attention to the frequent development of swelling of the optic discs after fractures of the vault of the skull. In a group of thirty cases of this order as many as sixteen showed this condition of papillœdema, and it is important to note that in none of these was there obvious visual defect. Here, again, relief of the pressure, as by decompression or trephining, was successful, for in all the cases the swelling of the optic discs disappeared quickly after the operation was performed. From these experiences it is evident that some of the anxious aspects of gunshot wounds of the skull depend upon an increase of intracranial pressure. Fortunately, such a condition is readily met by operation, but whether in the exigencies of the modern battlefield this can always be applied with sufficient promptness is another question. The respiratory and vagal centres are delicate mechanisms, and their collapse under sustained pressure cannot be long delayed. Mere artificial respiration may be sufficient when these centres are simply "stunned," as by a blow on the skull, but when there is intracranial effusion or hæmorrhage this must be removed if the respiratory centre is not to fail under increased pressure.

Another point which was enforced with practical unanimity by the speakers was the necessity for the removal of foreign bodies and for the thorough cleansing of the wound made by the bullet. The foreign bodies include, of course, not only the bullet, but also fragments of bone which may have been driven into the brain while the bullet itself remains on the surface. A very vigorous protest was entered against the tendency to "leave head cases alone." On the contrary, the general opinion seemed to be that both early and vigorous interference was necessary, and this with the object both of saving life and of restoring function. Certainly some of the cases related presented a very encouraging character so far as immediate results are concerned, and this obviously is the first step towards the desired end. Later consequences must be studied and dealt with as they arrive, and it is to be hoped that the Medical Society will adopt the suggestion, made by one of the speakers, to the effect that at some future date a discussion should be arranged on the late or postponed effects of gunshot injuries to the skull. There are, as is well known, many unhappy experiences in which, though life has been preserved, the disturbance of brain function has remained, and has manifested itself in various distressing fashions. One speaker contended that such experiences were largely due either to abstinence from operative interference or to delayed and ineffective surgery. Quasi-epileptic seizures following brain injuries have not proved very susceptible of relief by operation in the past, and perhaps it is not well to be too confident of the future.

